# A Perspective on Wearable Sensor Measurements and Data Science for Parkinson’s Disease

**DOI:** 10.3389/fneur.2017.00677

**Published:** 2017-12-12

**Authors:** Ricardo Matias, Vitor Paixão, Raquel Bouça, Joaquim J. Ferreira

**Affiliations:** ^1^Champalimaud Research, Champalimaud Centre for the Unknown, Lisbon, Portugal; ^2^Escola Superior Saúde – Instituto Politécnico de Setúbal, Setúbal, Portugal; ^3^Faculty of Medicine, Clinical Pharmacological Unit, Instituto de Medicina Molecular, University of Lisbon, Lisbon, Portugal; ^4^Campus Neurológico Sénior (CNS), Torres Vedras, Portugal

**Keywords:** Parkinson’s disease, wearable sensors, data science, biomarkers, biomechanics, clinical decision-making, decision support, motor symptoms fluctuations

## Abstract

Miniaturized and wearable sensor-based measurements enable the assessment of Parkinson’s disease (PD) motor-related features like never before and hold great promise as non-invasive biomarkers for early and accurate diagnosis, and monitoring the progression of PD. High-fidelity human movement reconstruction and simulation can already be conducted in a clinical setting with increasingly precise and affordable motion technology enabling access to high-quality labeled data on patients’ subcomponents of movement (kinematics and kinetics). At the same time, body-worn sensors now allow us to extend some quantitative movement-related measurements to patients’ daily living activities. This era of patient movement “cognification” is bringing us previously inaccessible variables that encode patients’ movement, and that, together with measures from clinical examinations, poses new challenges in data analysis. We present herein examples of the application of an unsupervised methodology to classify movement behavior in healthy individuals and patients with PD where no specific knowledge on the type of behaviors recorded is needed. We are most certainly leaving the early stage of the exponential curve that describes the current technological evolution and soon will be entering its steep ascent. But there is already a benefit to be derived from current motion technology and sophisticated data science methods to objectively measure parkinsonian impairments.

## Motor Symptoms Assessment in Parkinson’s Disease (PD)

Parkinson’s disease is the second most common neurological disorder, caused by the progressive loss of dopaminergic and other subcortical neurons. It is traditionally featured by its motor symptoms, hence its diagnosis is clinical, dependent on the presence of bradykinesia, which is associated with rest tremor, rigidity, and postural instability (1–3).

The classic motor features of PD typically start insidiously and are unilateral and mild, the response to treatment being excellent (3–5). 2–5 years after disease onset, the majority of patients experience wearing-off symptoms, involuntary movements, and other motor complications. Gait and balance disturbances, as well as speech and swallowing difficulties commonly appear, and response to treatment is only partial (5–8). After 10 years or more, most patients have developed a clearly bilateral disease, OFF states are associated with high disability and dependency, and ON states are also not good. Falls are common, and an increasing number of patients become at least temporarily wheelchair-bound. Dysarthria has a great impact on patients’ condition, and hinders communication with caregivers, whereas dysphagia is a frequent cause of choking and aspiration pneumonia, sometimes requiring tube feeding (6, 9).

Parkinson’s disease clinical assessment involves subjective patient reports of any changes in status since the last consultation and office-based assessments through clinical scales and traditional patient-reported outcomes (10–12). The use of standardized assessment tools in clinical practice and research is of utmost importance to assess disease progression, evaluate the effect of therapeutic interventions, and to communicate among colleagues (13). The most used instruments for PD assessment are the International Parkinson and Movement Disorder Society Unified Parkinson’s disease rating scale (MDS-UPDRS), to evaluate the presence, severity, and progression of PD symptoms, and the Hoehn and Yahr scale, which uses severity levels to evaluate disease progression (10, 11). However, numerous other tests and rating scales have been used, but there is not a consensus on the most suitable screening tools or monitoring outcomes (10, 11, 14).

Parkinson’s disease is notorious for its plethora of motor and non-motor features and for considerable inter and intra-subject clinical variability in clinical symptoms, disease progression, and response to medication. This usually reduces clinical visits to brief snapshots of patients’ health condition that not always reflects their real health-care status (11, 12, 15, 16). From a scientific and methodological perspective, the current standards of PD clinical evaluations have some limitations: (1) assessments requiring concentration and recall (e.g., fall diaries) are compromised by cognitive impairment, present in 80% of patients in the advanced stage of the disease; (2) the assessment is dependent on clinicians’ expertise and individual training; and (3) standard assessments are time consuming and location dependent (7, 11, 15). To obtain an accurate picture of symptoms, a continuous evaluation for prolonged periods of time is, therefore, required (11, 15).

There is a growing awareness that wearable technology, with its ability to capture movement continuously over longer periods of time in controlled and free-living environments, may overcome many of these limitations. It allows a higher sensitivity, accuracy, and reproducibility, and makes it more feasible to objectively capture the full complexity and diversity of changes in motor and non-motor behaviors (12, 15). Therefore, it not only answers to the difficulty to evaluate reliably fluctuating or rare events that, by definition, take place outside clinical visits (i.e., in daily life) but is also able to remotely capture behavioral data and use it to optimize treatment strategies through closed-loop systems (11, 12). Moreover, the use of wearable technology may have an impact on future clinical trials. It may have a significant impact on disease-modifying agents research, since it may offer a way to detect more readily subtle changes that were missed until now. Additionally, with wearable devices’ ability to measure outcomes at multiple time points, the statistical power of clinical trials can be enhanced and thus the sample size required to evaluate the effect of therapeutic interventions is reduced (12, 17).

With recent technological advances, a single worn device has the potential to provide a comprehensive picture of the patient within one assessment. A single sensor can quantify macro features, such as walking, sleeping, or sedentary time. It can also be broken down to detect very discrete features (micro features), such as a fall, gait characteristics, turning, and freezing (15). However, despite the variety of commercially available low-cost devices, the use of wearable technology in health care has not yet been established, since algorithm development and data analysis have not kept pace with sensor technology and design advances (15). Also, it is not enough to show that sensor-based technology can measure PD-related features. It is necessary to prove that those features are clinically relevant to patients and clinicians and useful to PD monitoring assessment. A measure is justified if it enhances our understanding of a complex disease or carries the potential to improve disease management as to the need and dose of therapy (11, 12). In order to distinguish relevant from futile technological-based outcome tools, researchers would need to determine: (1) which constructs are clinically relevant; (2) which contribute to an ecologically effective therapeutic decision and provide adequate information about a treatment response or disease course, and finally; (3) which allow an easy and repetitive use (12).

## PD Patient Biomechanical Analysis

Biomechanists and mobility researchers have seen human movement analysis evolve from a stick figure representation (18) to accurate real-time 3D movement reconstruction and simulation (19, 20). While such tools support many of today’s biomechanical laboratories’ services and research, some barriers (e.g., equipment costs and expertise) inhibit their use in clinical practice, and as a result, observation persists as the basis for patient movement analysis.

An increasing body of literature from a range of movement dysfunctions indicates that gait analysis with 3D movement measurements allows a more accurate assessment of gait deviations than visual-based gait analysis (21, 22) and supports its clinical efficacy (23). Musculoskeletal models (19, 24) can be used in clinical settings together with motion capture technology to accurately quantify 3D kinematic variables and complement (or even substitute) observational analysis. Being able to systematically quantify subcomponents of movement (including spatiotemporal variables, joint angles, angular velocity and acceleration, among others) is necessary to ascertain the motor state and monitor patients’ specific response to therapeutic interventions (25) and help diminish clinicians’ different rating strategies (26). Additionally, clinicians may want to know what were the forces and moment of force for each joint involved in the observed movement (27). This can be indirectly determined from the kinematics, external forces (if applicable), and model inertial properties by a process known as inverse dynamics. In gait analysis, external forces such as the ground reaction forces are collected using force platforms. Recent published literature is paving the way for predicting ground reaction forces based on measured kinematic data (28) only and by means of artificial neural networks (29). Such solutions will give clinicians access to new subcomponents of movement that are difficult or impossible to measure experimentally and may help overcome the costs and limitations of stationary devices like force platforms. Finally, simulations of human movement (forward dynamics) can be used to help understand what the kinematic results are through a set of given muscle activations. Data from these simulations provide clinicians with a cause-effect framework for analyzing the deviation of a patient’s movement pattern from the healthy pattern or from his movement pattern in the past. This type of framework can be used to answer “what if?” questions *via in silico* experiments and help plan therapeutic interventions targeting motor learning and control. Clinicians may access freely available full-body musculoskeletal models for simulations of human gait (30) and use them to synthesize movement patterns with minimum or even absence of experimental data in order to optimally perform a given movement task (often called predictive simulations) (31).

Optical motion capture systems are among the most commonly used solutions for human movement analysis in research laboratories. These systems are used to collect three-dimensional coordinates of special markers (and sometimes clusters) that are placed over the skin of one or more anatomical segments. These segments are tracked throughout the movements and sampled many times per second. To transform the markers’ coordinates into body segments’ kinematics (e.g., position and orientation), a multi-body chain where each anatomical segment is assumed as a rigid body is generally used. Because skin movements induce displacements in the position of the markers relative to the underlying bones, optimization methods need to be considered to improve segments’ pose estimation accuracy in each time frame (32). A new generation of more affordable optical motion capture systems is emerging, proving to be sufficiently accurate and reliable for use in clinical practice (33, 34) and providing a unique opportunity for patients’ movement analysis (35, 36) in clinical settings.

One of the major disadvantages of the optical motion capture systems is the limited workspace from where patients’ movement can be collected and the time needed for subject instrumentation. Recent advances in microelectromechanical systems provide a new generation of inertial measurement units (IMUs), giving a new surge to human movement analysis clinical and research communities. This new solution has a theoretically unlimited workspace, is cost-effective, and can be successfully used for accurate, non-invasive, and ambulatory motion tracking (37). An independent evaluation of market-available solutions corroborates that low cost and portable IMUs are an attractive solution for patients’ evaluation in a clinical setting, when compared to reported accuracy and reliability of optical motion capture systems (38).

Inertial measurement units are probably the most promising candidate for patients’ real-life mobility evaluation, extending and complementing the quantitative movement analysis performed in the clinical setting. In line with this, the development of new algorithms for classification of PD patients’ motor symptoms fluctuations based on a single IMU has received the attention of many researchers, and promising preliminary results have been published [e.g., Ref. (39)].

As stressed by the Movement Disorders Society Task Force on Technology (12), there is a strong need for a centralized, easy-to-access, and user-friendly platform, capable of merging clinical scores and outcomes, and the biomechanical-related information generated both in the clinical setting and during patients’ activities of daily living. With such a myriad of data centralized, sophisticated data science methods can be used to distil the data into knowledge and help support clinical decision-making, as elegantly summarized by Kubota and colleagues (40).

## Quantifying Movement Behavior in Patients with PD

In 1959, Arthur Samuel stated that “programming computers to learn from experience should eventually eliminate the need for much of this detailed programming effort” (41). Despite this promising claim, researchers that develop and use machine learning pipelines in their research know that it is still a tedious process whose performance depends on manually engineered features or hyperparameter settings and that requires a considerable degree of expertise and programming effort. While researchers are still tackling this and other bottlenecks to reduce the requirement of expert input to a minimum (42), the usage of machine learning has enabled the possibility of detecting new behaviors that were missed by traditional scoring methodologies and may soon allow us to describe and measure the complete behavioral repertoire of a patient (43). This, in turn, will empower clinicians and researchers to correlate the various features of behavior with collected patient data, instead of predicting which particular traits of behavior should be studied. To see how this could be achieved, it is important to understand how behavioral “classifiers” are developed using modern quantitative tools for measuring, describing, and analyzing behavior.

Methods designed to identify predefined blocks of motor behavior are in general incapable of discovering novel ones or new ways in which actions may be executed. One important characteristic of a behavior quantification algorithm should be the capacity of describing the behavior repertoire in its totality, including behaviors not anticipated by the researcher (44). As mentioned, these algorithms must determine explicit intervals of time when a relevant pattern of movement is executed (i.e., an action). These patterns (e.g., walking, running, or sitting) are detected by classifiers: computer algorithms that map input data to a category. A classifier can also distinguish occurrences of a specific action from periods where the action does not occur.

There are two distinct ways to train a classifier: supervised and unsupervised. In a supervised classifier, the behavior blocks are trained by “positive” (where the desired action happens) and “negative” templates (when it does not take place). The distinct actions are recognized by a machine learning algorithm that uses annotated training examples (“ground-truth” labels) to generate a set of rules to discriminate the different actions (44–47). In an unsupervised classifier, on the other hand, no previous assumptions are made about what type of behaviors are occurring. Only the representative templates with movement information without annotations are presented to the learning algorithm. The algorithm then groups data by its intrinsic structure into discrete units or blocks. The unsupervised classification is driven by the unlabeled data and the result of the procedure used as classifier labels for different sessions and subjects. Using this method, it is possible to generate a continuous unbiased classification of behavioral states.

**Figure 1:**
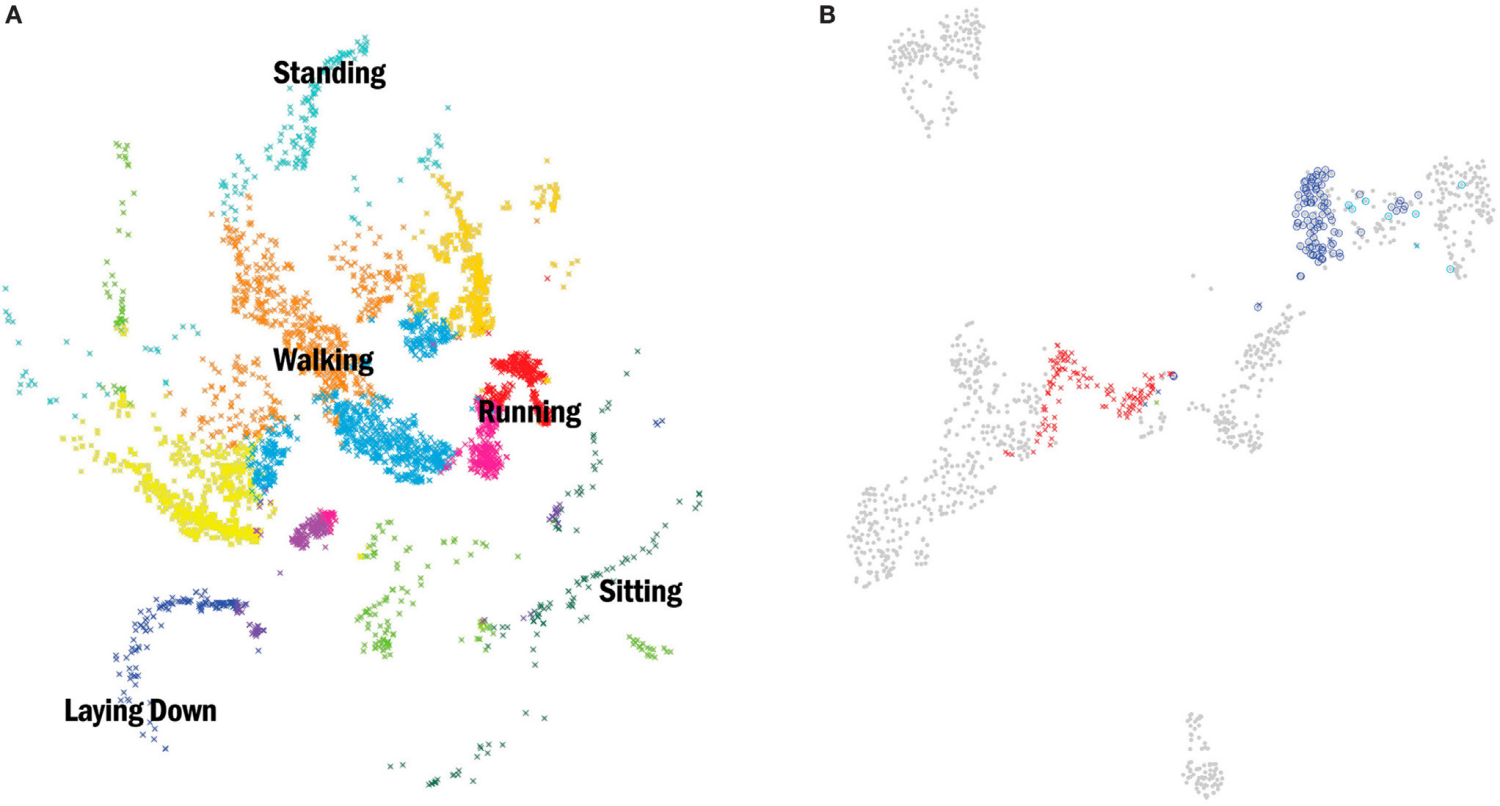
**(A)** (Left) two-dimensional embedding (t-Distributed Stochastic Neighbor Embedding) (50) of acceleration data points from nine healthy subjects performing several daily activities. Data were collected with one inertial measurement unit (IMU) placed over the pelvis with a sample rate of 120 Hz. Each point corresponds to a discretized behavior block obtained with a change-point detection algorithm. Colors correspond to clusters obtained by affinity propagation (51). A subset of clusters was labeled according to the dominant feature (standing, walking, sitting, etc.). **(B)** (Right) shows an example of the same methodology now applied to six patients diagnosed with idiopathic Parkinson’s disease (PD) and a Hoehn and Yahr stage less than or equal to 2. Movement data were collected with one IMU placed over the pelvis (sample rate 120 Hz) while patients walked a 10-m corridor, first in their OFF state (“x”) and later in their best ON state defined by the Movement Disorder Society Unified Parkinson’s disease rating scale (“o”). The gray dots represent all the recorded blocks of behavior from the six PD patients and the color dots the groups related to one patient. Clusters represented in both left and right images are from two independent experiments, thus, the resultant embeddings are not comparable.

**Figure 2:**
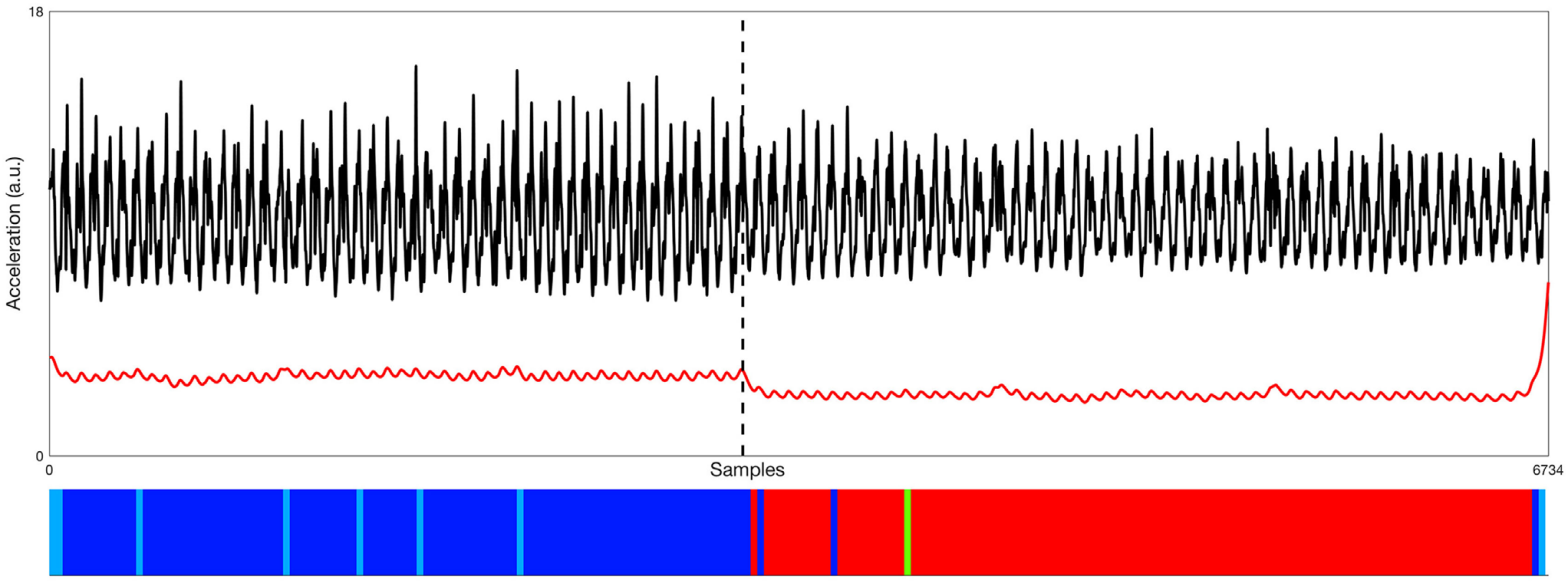
Representative example of data processed from the accelerometer (black signal—raw vertical acceleration; red signal—low-pass filtered posterior–anterior acceleration) used by one of the six Parkinson’s disease (PD) patients during two 10-m walk trials, first in his OFF state and later in his best ON state. Time series are two collated non-consecutive segments of data recorded OFF (left) and best ON (right) states of one PD patient, as indicated by the black dashed line. Bottom: the distinction between the OFF and ON states is very clear with this methodology, where the clusters’ organization (showed by the color bar) perfectly aligns with the transition of the signals above. Different colors represent found behavioral blocks (same color code as Figure 1B).

In the case of PD patients, where disturbance of motor activities rhythmicity is commonly present, mean measures will simply result in averaging out any modifications and may, therefore, be entirely insensitive. Clinicians and researchers should explore all subcomponents of movement and identify those that highlight non-constancy over multiple repetitions and prove to accurately measure variability (48, 49). In the following figures, we present examples of the application of movement behavior classification in healthy individuals (Figure 1A) and patients with PD (Figures 1B and 2) with the use of an unsupervised methodology.

The unsupervised clustering approach allowed us to generate a continuous unbiased classification of behavioral states in these examples with healthy individuals and PD patients, even without specific knowledge on the type of behaviors recorded (unlabeled data). In some cases, the found behavior blocks cannot be labeled, either because motor activities are impossible to observe or unknown altogether. Nevertheless, being able to categorize and discriminate these putative actions is important to describe the subject’s interaction with the environment, more importantly, so in ambulatory conditions and for long periods of time when direct access to the patient may not be possible (difficult).

Temporal variables that encode patients’ movement acquired both in a clinical setting and during patients daily living activities are often accompanied by structured covariates, such as patient demographics and measures from clinical examinations. In most cases, there are interactions and correlations between these movement-related variables and covariates that clinicians would like to understand and leverage. Fiterau and colleagues (52) recently presented a method that incorporates structured covariates into time series deep learning and demonstrated how the method outperforms competing models. Such methods obviate the usually required extensive feature engineering and domain expertise to unveil data interactions and correlations and are becoming increasingly available.

## Conclusion

We believe that the technological evolution witnessed in the last decades will allow us to “look” at patients in an unprecedented way, not only because the measurement equipment needed is becoming more accessible (e.g., motion sensors and software) but also because the dedicated expertise once required (e.g., in the form of a staff biomedical engineer) to collect, process, and present the data is nowadays facilitated by highly user-friendly web-based applications leveraged by sophisticated data science methods. The ill-equipped clinician can now be assisted in translating sensors’ data into actionable information upon which clinical decisions may be supported. Some of this technology was and continues to be miniaturized and made wearable, leading to a real-time quantified self. This will enable patients with movement disorders to benefit from an analysis that has been limited to academic laboratories and state-of-the-art medical centers. While this patient movement “cognification” is gaining a considerable body of evidence, showing that specific features related to the movement dysfunction can be measured by body-worn sensors, it still needs to prove its clinical impact and usefulness through well-designed studies. Although statistically significant changes may often be seen in movement-related variables in studies aiming to measure change after a given intervention, it is particularly important to determine clinimetric properties, such as the minimum clinically meaningful change, to fully understand its clinical significance.

Machine learning automatic pipelines like the one presented in the previous chapter can be applied to process 3D movement data in a clinical setting and help quantify patterns in the patients’ posture, balance, and gait, complementing the information of rating scales such as the MDS-UPDRS. Additionally, from the outcome of the classification, as shown in the previous chapter, we can study the behavior repertoire in real-life scenarios, assessing the micro structure of the continuous movement and behavioral sequences, and quantify transitions and behavior variability, both in the long-term monitoring experiments and also in more conventional tasks. This is of particular interest to track PD motor symptoms fluctuations during patients’ daily living activities and help clinicians objectively support decision-making with respect to adjustments to medication type, dose, and timing that we believe will lead to more effective treatments, patient quality of life, and an overall reduction in care-related costs.

It is our belief that the successful integration of miniaturized and wearable sensor-based measurements and data science methods in daily clinical practice will be deeply dependent on a concerted effort of both the research and clinical communities on: (i) guidelines to develop clinically accepted technology-based tools; (ii) promoting research networks and data sharing politics so that others can confirm and extend published results; (iii) learning how data science can and should be applied; (iv) promoting a collective intelligence to exponentially advance the quality of the assessment and management of PD patients.

## Author Contributions

Conceived and designed the manuscript: RM and JF. Data processing and interpretation: RM and VP. Wrote the paper and critical review: RM, VP, RB, and JF.

## Conflict of Interest Statement

The authors declare that there are no commercial or financial relationships that could be construed as a potential conflict of interest.
